# Generation and analysis of expressed sequence tags in the extreme large genomes *Lilium* and *Tulipa*

**DOI:** 10.1186/1471-2164-13-640

**Published:** 2012-11-20

**Authors:** Arwa Shahin, Martijn van Kaauwen, Danny Esselink, Joachim W Bargsten, Jaap M van Tuyl, Richard GF Visser, Paul Arens

**Affiliations:** 1 Wageningen UR Plant Breeding, Wageningen University and Research Centre, P.O. Box 386, Wageningen, 6700 AJ, The Netherlands; 2 Netherlands Bioinformatics Centre, Geert Grooteplein 28, Nijmegen, 6525 GA, The Netherlands; 3 Applied Bioinformatics, Plant Research International, PO Box 619, Wageningen, 6700 AP, The Netherlands

**Keywords:** Flower bulb, Next generation sequencing, Gene ontology, SNP markers, SSRs, OrthoMCL, Comparative Genomics, Monocot

## Abstract

**Background:**

Bulbous flowers such as lily and tulip (*Liliaceae* family) are monocot perennial herbs that are economically very important ornamental plants worldwide. However, there are hardly any genetic studies performed and genomic resources are lacking. To build genomic resources and develop tools to speed up the breeding in both crops, next generation sequencing was implemented. We sequenced and assembled transcriptomes of four lily and five tulip genotypes using 454 pyro-sequencing technology.

**Results:**

Successfully, we developed the first set of 81,791 contigs with an average length of 514 bp for tulip, and enriched the very limited number of 3,329 available ESTs (Expressed Sequence Tags) for lily with 52,172 contigs with an average length of 555 bp. The contigs together with singletons covered on average 37% of lily and 39% of tulip estimated transcriptome. Mining lily and tulip sequence data for SSRs (Simple Sequence Repeats) showed that di-nucleotide repeats were twice more abundant in UTRs (UnTranslated Regions) compared to coding regions, while tri-nucleotide repeats were equally spread over coding and UTR regions. Two sets of single nucleotide polymorphism (SNP) markers suitable for high throughput genotyping were developed. In the first set, no SNPs flanking the target SNP (50 bp on either side) were allowed. In the second set, one SNP in the flanking regions was allowed, which resulted in a 2 to 3 fold increase in SNP marker numbers compared with the first set. Orthologous groups between the two flower bulbs: lily and tulip (12,017 groups) and among the three monocot species: lily, tulip, and rice (6,900 groups) were determined using OrthoMCL. Orthologous groups were screened for common SNP markers and EST-SSRs to study synteny between lily and tulip, which resulted in 113 common SNP markers and 292 common EST-SSR. Lily and tulip contigs generated were annotated and described according to Gene Ontology terminology.

**Conclusions:**

Two transcriptome sets were built that are valuable resources for marker development, comparative genomic studies and candidate gene approaches. Next generation sequencing of leaf transcriptome is very effective; however, deeper sequencing and using more tissues and stages is advisable for extended comparative studies.

## Background

Lily and tulip (*Liliaceae* family) are monocot perennial herbs that have unsurpassed beauty and great commercial significance. They are also very interesting from an evolutionary point of view since both species have very huge genomes (1C = 25 GB for tulip, and 36 GB for lily). The two species are comparable in several aspects: both are bulbous monocots, have 2n = 2x = 24 chromosomes, and a long growth cycle (2–3 years for lily and 5–6 years for tulip). For both species genetic resources are limited.

The genus *Lilium*, includes around 100 species which are taxonomically classified into seven sections: *Martagon*, *Pseudolirium*, *Lilium*, *Archelirion*, *Sinomartagon*, *Leucolirion*, and *Oxypetala*[1,2]. Different species within each section are relatively easy to cross and hybrids are fertile [3,4]. Hybrids within sections *Leucolirion*, *Archelirion*, and *Sinomartagon* represent the most important groups for breeding and are referred to as: Longiflorum (L), Trumpet (T), Asiatic (A), and Oriental (O) hybrids. An extensive number of cytogenetic studies explored karyotypes of lily e.g. [5-7]. Meiosis of interspecific hybrids and cytological maps of three complete genomes of lilies (L, A, O) based on recombination sites in BC progenies of two interspecific hybrids [8] were studied. On the other hand, genetic mapping of lily has not yet been well studied. The currently available genetic maps which were constructed using dominant markers (AFLP ‘Amplified Fragment Length Polymorphism’, NBS ‘Nucleotide Binding Site’, and DArT ‘Diversity Arrays Technology’) are not well saturated [9]. The available EST data [10] in the sequence database is very limited with only 3,329 ESTs deposited [10].

The genus *Tulipa* L. comprises about 100 species [11] that are taxonomically classified into two subgenera: *Tulipa* and *Eriostemones*[12,13]. Subgenus *Tulipa* is subdivided into five sections named: *Tulipa*, *Eichleres*, *Tulipanun*, *Kolpakowskianae*, and *Clusianae*. The commercial cut flower assortment of tulips consists mainly of cultivars from *Tulipa gesneriana* (section *Tulipa*) and *T. fosteriana* (section *Eichleres*) [14]. So far, there are no genetic maps or molecular markers published for tulip, and additionally no ESTs are found in the databases for this species.

*Lilium* and *Tulipa* are expected to be highly heterozygous species since both are outcrossing species and derived from a number of interspecific crosses. However there is no data available on the actual levels of heterozygosity within each species.

Breeding in these two species is limited by their long juvenile phase whereas the success of new cultivars is increasingly influenced by the presence of disease resistances against *Fusarium*, *Botrytis*, and tulip breaking virus [15,16]. These resistances are difficult to breed for using classical breeding because of the quantitative nature of the resistances and/or elaborate disease tests. For instance, *Fusarium* resistance in lily is known to be controlled by six putative QTLs (Quantitative Trait Locus) and disease tests are highly influenced by environment [9]. Developing user friendly, efficient, transferable, and co-dominant markers such as SNPs and SSRs markers that can be implemented in molecular assisted breeding (MAB) applications will help to speed up breeding in these two species.

Recent studies have shown that next generation sequencing technology can be an effective tool to generate huge amounts of sequence data in a short time which can be implemented in all types of genetic and genomic studies such as: transcriptome characterization, molecular marker development [17-19], ecological genetics [20], and evolutionary studies [21]. With the purpose of generating the first broad survey of genes in lily and tulip, we sequenced and assembled transcriptomes of four lily and five tulip genotypes using 454 pyro-sequencing. The sequence assemblies were used to identify a set of SNPs suited for high throughput genotyping purposes, and to screen for EST-SSRs. Orthologous genes between lily and tulip were identified and compared with the model species ‘rice’. The whole set of generated contigs for lily and tulip were annotated and described according to GO (gene ontology) terminology. Common markers that can be genotyped and mapped in both species were identified based on orthologous genes.

## Results and discussion

### EST sequencing and assembly

We performed 454 GS FLX Titanium pyro-sequencing on nine normalized cDNA libraries constructed from leaves of four lily genotypes (‘Connecticut King’, ‘White Fox’, ‘Star Gazer’, and ‘Trumpet 061099’), and five tulip genotypes (‘Cantata’, ‘Princeps’, ‘Ile de France’, ‘Kees Nelis’, and ‘Bellona’). The number of sequenced reads obtained varied between 139,480 reads for ‘Connecticut King’ and 592,034 reads for ‘Kees Nelis’ (Table 1). The percentage of sequence reads that was retained for assembly after quality filtration ranged between 67% and 75% (Table 1) which was somewhat higher than those for 454/Sanger data of *Eucalyptus* (60.7%) [22], and close to the 79% for *Pinus contorta*[23]. Average read length ranged between 278 bp for ‘Bellona’ and 389 bp for ‘Cantata’ (Table 1). These results were comparable (and even better in some genotypes) with that obtained in other studies like Blanca et al. [17] where the processed reads of cucurbit retained after trimming was 64% with an average read length of 321 bp. After filtration, remaining reads were used for *de novo* assembly using CLC.

**Table 1:** General statistics of 454 sequencing and assembly for lily and tulip

**Genotype**	**Nr. reads**	**Nr. reads after filtration**	**Avg. read length bp**	**Nr. assembled reads**	**Singletons**	**Nr. contigs**	**Avg. EST length bp**
**Connecticut King**	139,480	104,323(75%)	336	77,097(74%)	27,226(26%)	14,773	615
**White Fox**	326,539	221,597(68%)	338	182,393(82%)	39,204(18%)	21,898	663
**Star Gazer**	374,240	255,081(68%)	341	202,707(79.5%)	52,374(20.5%)	24,700	688
**Trumpet**	442,476	299,655(69%)	343	241,782(81%)	57,873(19%)	26,075	694
**Lily-All**	1,282,735	880,656(69%)	340	471,378(53.5%)	409,278(46.5%)	52,172	555
**Cantata**	310,973	207,229(67%)	389	158,007(76%)	49,222(24%)	17,646	625
**Princeps**	316,372	211,380(67%)	386	165,282(78%)	46,098(22%)	17,007	632
***T. fosteriana***	627,345	418,609(67%)	388	293,043(70%)	125,566(30%)	24,713	629
**Kees Nelis**	592,034	407,392(69%)	281	303,558(74.5%)	103,834(25.5%)	38,716	559
**Ile de France**	263,175	185,464(70%)	283	125,293(67.6%)	60,171(32%)	24,557	517
**Bellona**	221,334	149,768(67%)	278	109,309(34%)	40,459(27%)	14,325	522
***T. gesneriana***	1,076,543	742,624(69%)	281	536,776 (74%)	205,848(28%)	54,575	557
**Tulip-All**	1,703,888	1,378,898	314	827,772(60%)	551,126(40%)	81,791	514

Currently, a total of 3,090 lily’s ESTs are available in the nucleotide sequence databases generated from *Lilium formosanum* (1324) [10], *L. longiflorum* (991), Oriental hybrids (565), and *L. regale* (210). These ESTs could be clustered into just 381 contigs [24]. In this study, we generated 52,172 consensus sequences (non-redundant sequences or contigs) representing gene fragments from the four main groups of *Lilium*. Also, 81,791 contigs for tulip, representing the two main groups of commercial tulips: *T. fosteriana* and *T. gesneriana*, were generated which present the first EST data for tulip. Overall, the number of lily contigs generated in this study is comparable to that obtained in other transcriptome analyses such as for cucurbit (49,610 contigs; two cultivars) [17], and for *Eucalyptus* (48,973 contigs; six species) [22]. The number of tulip contigs is at the high end. It is, however, important to keep in mind that number of generated contigs does not reflect number of genes. Fragments of one gene could be assembled in different contigs due to: short contigs length (range of 500 to 700 bp) compared with the average gene length (2 Kb), missing overlap among contigs which might be related to the not fully unbiased cDNA synthesis step in sequence library construction using random hexamer primers, or orthologous sequences among genotypes are assembled into different contigs due to high genetic divergence among different genotypes.

Running assembly for the four lily genotypes together (Lily-All assembly) or for the five tulip genotypes together (Tulip-All) resulted in a dramatic increase in singleton and contig numbers (Table 1). These effects can be explained because different sets of genes were being sequenced among the different genotypes, and/or that orthologous sequences among genotypes tend to split up into different contigs due to the high level of heterogeneity among the genotypes [24]. For lily, the four genotypes were a result of interspecific crosses between different species within their respective sections. In tulip, there is a slightly similar situation for the difference between *T. gesneriana* and *T. fosteriana*. The fact that the assembly of reads from the tulip genotypes within their respective *T*. *gesneriana* and *T*. *fosteriana* sections shows a much better performance confirms the influence of heterogeneity in the assembly.

CLC assembler with default setting was used to assemble lily and tulip data since it showed to be capable to handle sequence data of heterozygous nature more efficiently compared with other assemblers like: CAP3, MIRA, Velvet, and SOAP regarding number of contigs, number of singletons, and redundancy [24-26]. The parameters of CLC were not tested further as using less stringent parameters might lead to an increase of chimeric contigs due to the assembly of paralogs in one contig [24]. Absence of a complete genome sequence for lily and tulip, or for a close relative, makes it difficult to check the most optimal assembly settings with respect to the quality of assembly. Consequently, Lily-All and Tulip-All assemblies were not used for markers development to avoid possible mistakes related to the assembly of these relatively distant genotypes. Instead orthologous groups determined by OrthoMCL were used for marker development between different genotypes (common markers).

An estimation of transcriptome coverage of lily and tulip genotypes was made (Table 2). There is no information about the total size or number of genes in lily and tulip. Therefore, transcriptome size was assumed to be similar to that of rice, which is also a monocot species. The gene space of rice was estimated to be around 82 Mb (41,000 genes with an average gene length of 2 Kb, [27]). Gene coverage for each lily and tulip genotype was calculated based on total number of bases generated (assembled sequences and singletons) as a percentage of the assumed gene space (82 Mb). In lily, gene coverage varied between 26% in ‘Connecticut King’ and 46% in ‘Trumpet’, with average gene coverage of 37%. In tulip, gene coverage was on average 39%, varying from 23% in ‘Bellona’ to 63% in ‘Kees Nelis’. The combined *T. gesneriana* cDNA sequences seem to cover the entire gene space although two-thirds was derived of singletons (Table 2). The large number of contigs generated and good coverage of the transcriptome for both species shows the high efficiency of next generation sequencing technology, especially taking into account that a single 454 run of normalized cDNA libraries, constructed out of one tissue and from a single growing stage was used. However to further improve transcriptome coverage, sequencing different tissues and developmental stages is needed.

**Table 2:** Transcriptome coverage of lily and tulip genomes

**Genotype(s)**	**Assembled Sequences (MB)**	**Singletons (MB)**	**Total (MB)**	**Transcriptome Coverage %**
**Connecticut King**	10	11.2	21.2	25.8
**White Fox**	14.5	13.2	27.7	33.8
**Star Gazer**	17	17.9	34.9	42.6
**Trumpet**	18	20	38	46.3
**Cantata**	11	19.5	30.5	37.2
**Princeps**	10.8	18	28.8	35
***T. fosteriana***	16	50.7	66.7	81.3
**Kees Nelis**	21.6	30	51.6	63
**Ile de France**	12.7	17	29.7	36
**Bellona**	7.5	11	18.5	22.6
***T. gesneriana***	30.4	60	90.4	110

The estimated percentage of transcriptome coverage for each genotype was calculated based on the number of genes and average gene size in rice.

### SNP marker detection

Contigs that contain at least one SNP (the two different nucleotides were present in at least two independent reads each) were identified using QualitySNP [28] software and their percentage of the total contig number was calculated (Table 3). This percentage exceeded 40% for lily genotypes except for ‘Connecticut King’ (Table 3). Similarly in tulip, this percentage also exceeded 40% in *T. fosteriana*, while it was lower in *T. gesneriana* genotypes (Table 3). These results were comparable to those detected in other outcrossing species like *Eucalyptus* (40%) [22].

**Table 3:** SNP markers identification for lily and tulip

**Genotype(s)**	**Nr. contigs**	Nr. contigs containing at least one SNP*	**Nr. SNP markers**	**Nr. SNP markers**
			(no secondary SNP) **	(one secondary SNP) **
**Connecticut King**	14,773	4,309 (29%)	406 (9.4%)	1,171 (27%)
**White Fox**	21,898	9,261 (42%)	558 (6%)	1,292 (14%)
**Star Gazer**	24,700	10,024 (41%)	730 (7%)	2,026 (20%)
**Trumpet**	26,075	11,298 (43%)	607 (5%)	2,075 (18%)
**Cantata**	17,646	7,456 (42%)	722 (10%)	2,371 (32%)
**Princeps**	17,007	7,587 (45%)	690 (10%)	2,510 (33%)
***T. fosteriana***	24,713	11,787 (48%)	1,002 (8.5%)	3,265 (28%)
**Kees Nelis**	38,716	13,832 (36%)	595 (4.3%)	1,646 (12%)
**Ile de France**	24,557	6,347 (26%)	310 (5%)	776 (12%)
**Bellona**	14,325	4,476 (31%)	223 (5%)	535 (12%)
***T. gesneriana***	54,575	20,661 (38%)	822 (4%)	2,033 (10%)

* Percentages of contigs that contain at least one SNP calculated according to the total number of contigs.

** Percentage of SNP markers from total nr. of contigs that contain at least one SNP.

Number of contigs that contain at least one SNP was calculated. SNP markers with 50 bps flanking sequences free of secondary SNP, and with one secondary SNP allowance in the flanking sequences were identified.

QualitySNP [28] software was also used to identify single nucleotide polymorphisms that can be used as SNP markers by comparing reads within each contig. We analyzed only SNPs and excluded all InDels due to the fact that 454 has serious problems with mono-nucleotide tracts and may introduce InDels without biological significance frequently.

Two sets of SNP markers were developed. The first set consisted of markers that have no other SNPs in the 50 bp flanking regions of the target SNP. The percentages of these markers compared to total number of contigs that have at least one SNP were calculated (Table 3). The highest percentage in lily was for ‘Connecticut King’ (9.4%), while the other three cultivars showed lower percentages (around 6%). In tulip, the percentage of SNP markers for *T. fosteriana* cultivars (10%) was two times higher than for *T. gesneriana* genotypes (5%). The second set of SNP markers also allowed markers that have one secondary SNP in the 50 bp flanking regions, which caused a 2 to 3 times increase in the number of SNP markers (Table 3). The number of SNP markers identified in each genotype then ranged between 1,171 and 2,075 SNP markers in lily and between 535 and 2,510 SNP markers in tulip. Compared with the 572 SNP markers generated in *Eucalyptus* when no control on the flanking SNPs was applied [22] this indicates that the heterozygosity of both bulbous crops is considerable.

### Mining for microsatellites

We screened lily and tulip contigs for the presence of SSRs, and analyzed their nature and frequency (Table 4). Percentages of EST-SSR (compared to the total number of contigs) found in lily genotypes were comparable with each other (around 2.7%) except for ‘Connecticut King’ that showed a lower percentage (1.9%). In tulip, percentages of EST-SSR in contigs were similar within *T. fosteriana* genotypes (‘Cantata’ and ‘Princeps’, around 4%), and similar within *T. gesneriana* genotypes (‘Bellona’, ‘Ile de France’, and ‘Kees Nelis’, around 2%), although lower in *T. gesneriana* compared to *T. fosteriana*. Having the same criteria for SSR retrieval, the percentages of SSRs found for lily and tulip were higher than results found for *Medicago truncatula* (0.2%) [29], comparable to grape and barley (3 and 2.8%, respectively) [18,30], and lower than for pigeon pea (7.6%) [31].

**Table 4:** SSR repeat description in lily and tulip

**SSR motif**	**Nr. contigs**	Total Nr. SSR*	di-**	Tri-**	**Tetra-**	**Penta-**	**Hexa-**
**Connecticut King**	14,773	271 (1.9%)	85 (31%)	161 (59%)	4	6	15
**White Fox**	21,898	603 (2.8%)	216 (36%)	301 (50%)	51	12	23
**Star Gazer**	24,700	735 (3%)	299 (41%)	330 (45%)	66	13	27
**Trumpet**	26,075	745 (2.8%)	312 (42%)	341 (46%)	50	17	25
**Cantata**	17,646	696 (3.9%)	168 (24%)	449 (65%)	30	9	40
**Princeps**	17,007	683 (4%)	146 (21%)	468 (69%)	28	11	30
***T. fosteriana***	24,713	957 (3.9%)	216 (23%)	642 (67%)	45	15	39
**Kees Nelis**	38,716	881 (2.3%)	262 (30%)	491 (56%)	58	19	51
**Ile de France**	24,557	521 (2%)	140 (27%)	317 (61%)	33	12	19
**Bellona**	14,325	302 (2%)	80 (28%)	184 (64%)	9	11	18
***T. gesneriana***	54,575	1,302 (2.9%)	393 (30%)	719 (55%)	95	35	60

* Percentage of SSR repeats calculated according to the total number of contigs.

** Percentage of SSR repeats from total nr. of SSRs.

Frequency and distribution of di-, tri-, tetra-, and hexa-nucleotide repeats were analyzed in each genotype (Table 4). In both species, the most frequent repeat motif was AG/CT for di-nucleotide repeats and CCG/CGG for tri-nucleotide repeats. Similar results were found in barley [32] which is also a monocot with a large genome size. More than 86% of the identified EST-SSRs in lily and tulip are di- or tri- nucleotide repeats. In lily, tri-nucleotide repeats were just slightly more abundant than di-nucleotide repeats although almost equal amounts were found in ‘Star Gazer’ and ‘Trumpet’ (Table 4). In tulip, tri-nucleotide repeats were around two fold more abundant than di-nucleotide repeats (Table 4). This finding in tulip is in agreement with previous findings in grape and castor bean [18,33].

Previous studies have shown a dominance of tri-nucleotide repeats in coding regions as can be expected because length variance for tri-nucleotide motifs does not result in frame shifts in genes [18]. Accordingly di-nucleotide repeats were found to be dominant in the 5′- and 3′-UTR regions [34]. Our analysis in lily and tulip showed a selection against di-nucleotide repeats in coding regions compared with UTR regions (Table 5). The percentage of di-nucleotide repeats in coding regions (32%) was half of that in UTR regions (68%), while tri-nucleotide repeats were spread with equal frequency over coding and UTR regions (Table 5). These results, are in line with the result in wheat [34].

**Table 5:** Location of di- and tri-nucleotide repeats in lily and tulip contigs presented as a percentage of the total number of identified SSR in each cultivar in coding and UTR regions identified using ORF-Predictor software

	**di-nucleotide repeats**		**Tri-nucleotide repeats**	
	**Coding region %**	**UTR regions %**	**Coding region %**	**UTR regions %**
**Connecticut King**	35	65	50	50
**White Fox**	28	72	44	56
**Star Gazer**	32	68	58	42
**Trumpet**	33	67	58	42
**Cantata**	32	68	57	43
**Princeps**	26	74	57	43
**Kees Nelis**	32	68	48	52
**Ile de France**	35	65	50	50
**Bellona**	43	57	52	48

### Orthologous sequences

**Figure 1:**
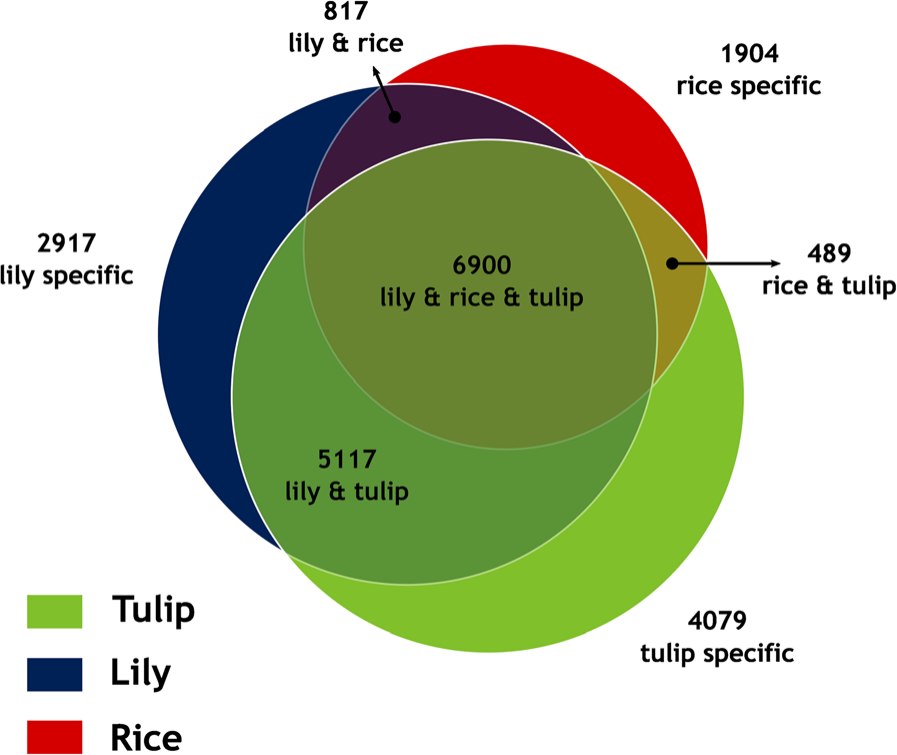
**Venn diagram of the distribution of orthologous groups in lily, tulip and rice, calculated with OrthoMCL.** Overlapping regions denote groups with at least two proteins of all species that are part of the intersection. All circles and overlapping areas are scaled to the number of groups in the respective region.

Having cDNA sequence data, allows the use of comparative genomics to reveal regions of sequence conservation [35] and hence improve our understanding of the species evolution. To define conserved genes between lily and tulip, and compare that with the most sequenced and annotated monocot species ‘rice’, orthologous groups that are shared among them were identified. Protein sequences of the rice genome (55,803 protein) were retrieved from Phytozome ( http://phytozome.net, [36]) for comparison. Contig sequences of the nine lily and tulip genotypes were translated using ESTscan2 [37,38] and compared with rice proteins using OrthoMCL [39]. A total of 255,500 protein sequences of rice, lily, and tulip were clustered into 22,223 orthologous groups. A total of 10,110 orthologous groups for rice, 15,751 orthologous groups for lily, and 16,585 orthologous groups for tulip were generated (Figure 1). Overall, 6,900 groups contained sequences from all three species, 817 groups were specific for lily and rice, 489 groups were specific for tulip and rice, and 5,117 groups were specific for lily and tulip (Figure 1). The 6,900 groups that are shared among the three monocot species represent 31% of the total number of orthologous groups identified in this study. This percentage is far less than the 71% shared orthologous groups among three monocot grasses species: rice, sorghum, and *Brachypodium*[40]. However, the divergence among rice, sorghum, and *Brachypodium* dated back 47 Myr ago [41], while the divergence between rice and *Lilium* or *Tulipa* is much older. It has been reported that the divergence between rice and *Musa* took places around 117 Myr ago [41,42] and between rice and *Allium* is more than 150 Myr ago [41]. Consequently, the divergence between rice (*Poaceae*) and *Lilium* and *Tulipa* (*Liliaceae*) is expected to be between 170–200 Myr ago [42] which explains the lower number of shared orthologous groups identified between rice and *Liliaceae* in our study.

The number of orthologous groups between lily and tulip (5,117 and 6900 groups, 54% of the total orthologous groups identified in this study) is less than the 67% shared orthologous groups between tomato and potato [43]. This low percentage of shared orthologous groups between lily and tulip might be related to the older divergence time (20 Myr) between members of *Liliaceae* family [44], compared with 7.3 Myr tomato-potato divergence [43]. However, we do expect that the percentage of shared orthologous groups will increase by sequencing more tissues and different developmental stages of the different genotypes.

### Gene annotation and gene ontology

For gene annotation we used the assemblies Lily-All and Tulip-All to survey what types of genes are present in both flower bulb species. Also the 6,900 orthologous groups from the OrthoMCL analyses were annotated to identify the type of genes that are shared among the three monocot species (lily, tulip, and rice).

A Blast analysis using the non-redundant protein database (nr) from NCBI with an E value threshold of 1E-15 was performed using Blast2Go software [45]. At least one significant blast hit was found for 49% of Lily-All contigs (25,385 contigs, Additional file 1), 30% of tulip-All contigs (24,704 contigs, Additional file 2), and 93% of the orthologous groups (6,900 groups, Additional file 3). As was expected, *Oryza sativa* (the most sequenced and annotated monocot species) showed to be the closest species to both lily and tulip because most first hits were with sequences from this species. Having only 49% and 30% of lily-All and tulip-All contigs annotated, respectively, demonstrates the very rich source of not yet identified genes that need to be annotated. However, not all genes in the genebank are annotated, and it is also possible that genes from lily and tulip deviate significantly at the sequence level from the existing orthologous genes in databases at the threshold value of 1E-15, or that the length of part of the contigs is not enough to find significant similarity.

Gene ontology provides a structured and controlled terminology to describe gene products according to three categories: molecular function (refers to a biochemical activity of a gene product without stating where or when the event happens), biological process (refers to a biological objective to which the gene product contributes), and cell component (refers to the place in the cell where a gene product is active) [46]. Since genes can be part of different pathways or have more than one function at the same time, the same gene can have more than one GO description (GO term) and thus belong to more than one of the earlier mentioned categories. The annotated contigs of Lily-All*,* Tulip-All, and the orthologous sequences among lily, tulip, and rice were used for gene ontology assignments. Gene ontology assignments of Lily-All contigs were divided into: 42% (molecular function), 31% (biological process), and 27% (cellular component). In Tulip-All contigs, gene ontology assignments were divided into: 19% (molecular function), 42% (biological process), and 39% (cellular component) contigs.

**Figure 2:**
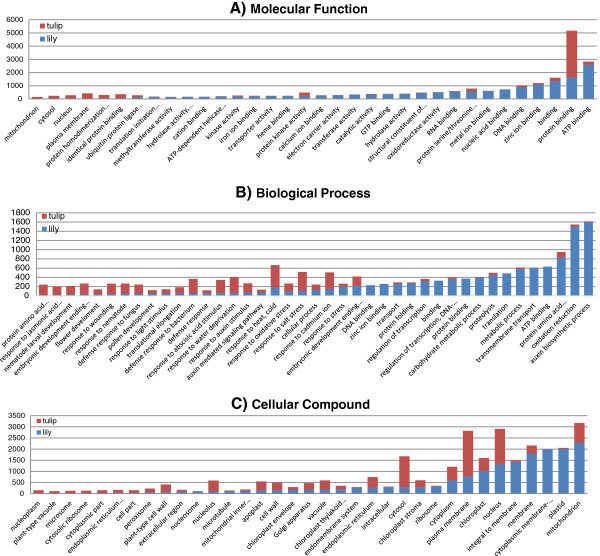
**Representation of transcriptome ontology assignments for Lily-All and Tulip-All assemblies of 454 sequencing data.** GO terms: **A**, molecular function; **B**, biological process; **C**, cellular compound.

Both species showed to have similar GO terms in the three categories. The differences were in the amount of contigs annotated for each GO term. In the category molecular function, the most represented GO terms were of binding function such as ‘protein binding’, ‘ATP binding’, ‘binding’, ‘nucleic acid binding’ in addition to all types of activities such as ‘protein kinase activity’, ‘transferase activity’, ‘transporter activity’, ‘catalytic activity’, and ‘oxidoreductase activity’ (Figure 2A). The GO terms that were identified in lily and tulip (Figure 2A) were identified as well in *Medicago truncatula*, *Cucurbita pepo*, *Cucurbita melo*, and *Oryza sativa*[17,29,47,48]. Ion binding terminology such as ‘calcium binding’, ‘iron binding’, and ‘zinc binding’ were highly represented in lily (Figure 2A), similar to olive leaf [49].

In the category biological process, there were clear differences between lily and tulip in the enrichment of GO terms (Figure 2B). Lily’s contigs were more concentrated in activities like ‘auxin biosynthetic process’, ‘oxidation reduction’, ‘metabolic process’, ‘carbohydrate metabolic process’, ‘translation’, ‘protein amino acid binding’, and ‘transmembrane transport’ whereas response to biotic and biotic stresses such as responses to salt, heat, cold, nematode, bacteria, virus, and fungus stresses were more represented in tulip (Figure 2B).

The GO terms ‘flower development’, ‘embryonic development’, and ‘pollen development’ are present in our data although we sequenced young leaves. This is either related to the combination of flowering and vegetative growing stages (mainly in tulip since its onset of leaf to seed formation is short (7–12 weeks)), or genes are involved in different pathways and not only in flower development. On the other hand, the high level of ‘auxin biosynthetic process’ recorded in lily might reflect the central on-going processes which are mainly plant-cell elongation, apical dominance (inhibit growth of lateral buds), and rooting processes which are all known to be controlled by auxin.

The GO terms of cellular compound category showed significant representation of ‘mitochondrion’, ‘plastid’, ‘plasma membrane’, ‘membrane’, ‘nucleus’, ‘cytosol’, ‘chloroplast’, and ‘integral to membrane’ (Figure 2C) which was similar to previous studies [17,47,50]. All contigs of mitochondria, chloroplast, and plastid that were defined here (Figure 2C), are very interesting for phylogenetic studies but may be less suitable for marker development aiming at mapping for breeding purposes.

**Figure 3:**
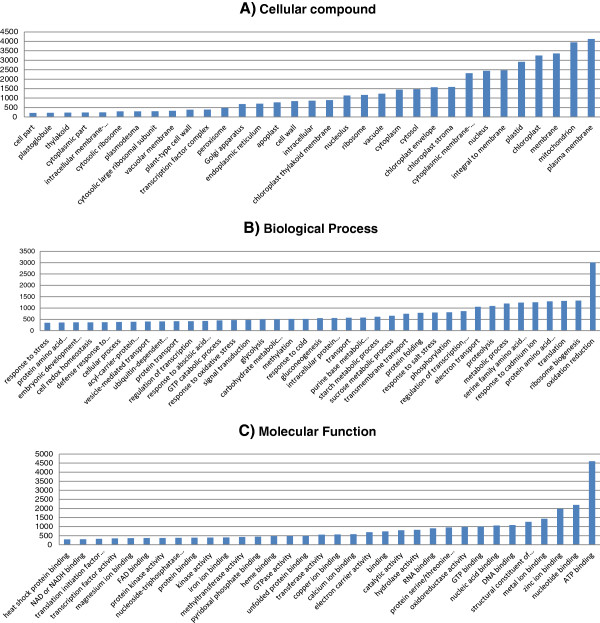
**Representation of transcriptome ontology assignments (GO term) for the orthologous sequences between lily and tulip from 454 sequencing data. A**, molecular function, **B**, biological process and **C**, cellular compound.

GO assessment of the 6,900 orthologous groups among tulip, lily, and rice were divided into: 31% (molecular function), 41% (biological process), and 28% (cellular component). A summary description of annotated contigs for the orthologous genes in each GO category is provided in Figure 3. Genes essential for growing and defense processes are shown to be the main orthologous sequences between the three species. Genes involved in response to biotic, abiotic, and endogenous stimulus were also defined (Figure 3B). Under molecular function category, mainly binding activity was identified (Figure 3C). Overall, the majority of orthologous genes were housekeeping genes. More detailed data has become available that can serve as a major resource for further research (Additional files 1, 2, 3). It is interesting to study the GO terms enrichment of orthologous groups specific for lily and tulip (5,117 groups, Figure 1) because they may consist of genes specific for bulbous crops.

### Identification of common SNP markers and SSRs within and between lily and tulip

Exchanging genetic information between two related species by linking their genetic maps would be of great interest. This linking will facilitate comparative mapping of genes across distantly related plant species by direct comparison of DNA sequences and map positions such as between wheat and barley, tomato and potato, and *Arabidopsis* and *Brassica*[51-53]. Identification of polymorphisms in orthologous sequences that allow marker development in both species will provide a set of common genetic loci that can be implemented for comparative mapping and thus improve our understanding of the evolutionary history (gene duplication, conversion, and rearrangement) of the lily and tulip genome. For this, SNP markers and EST-SSR were developed from the parents of mapping populations in lily (‘Connecticut King’ and ‘White Fox’) and tulip (‘Cantata’ and ‘Kees Nelis’). The orthologous groups identified by OrthoMCL were extracted for each of the four parents’ combinations. These orthologous groups were searched for SNP markers and SSRs.

**Figure 4:**
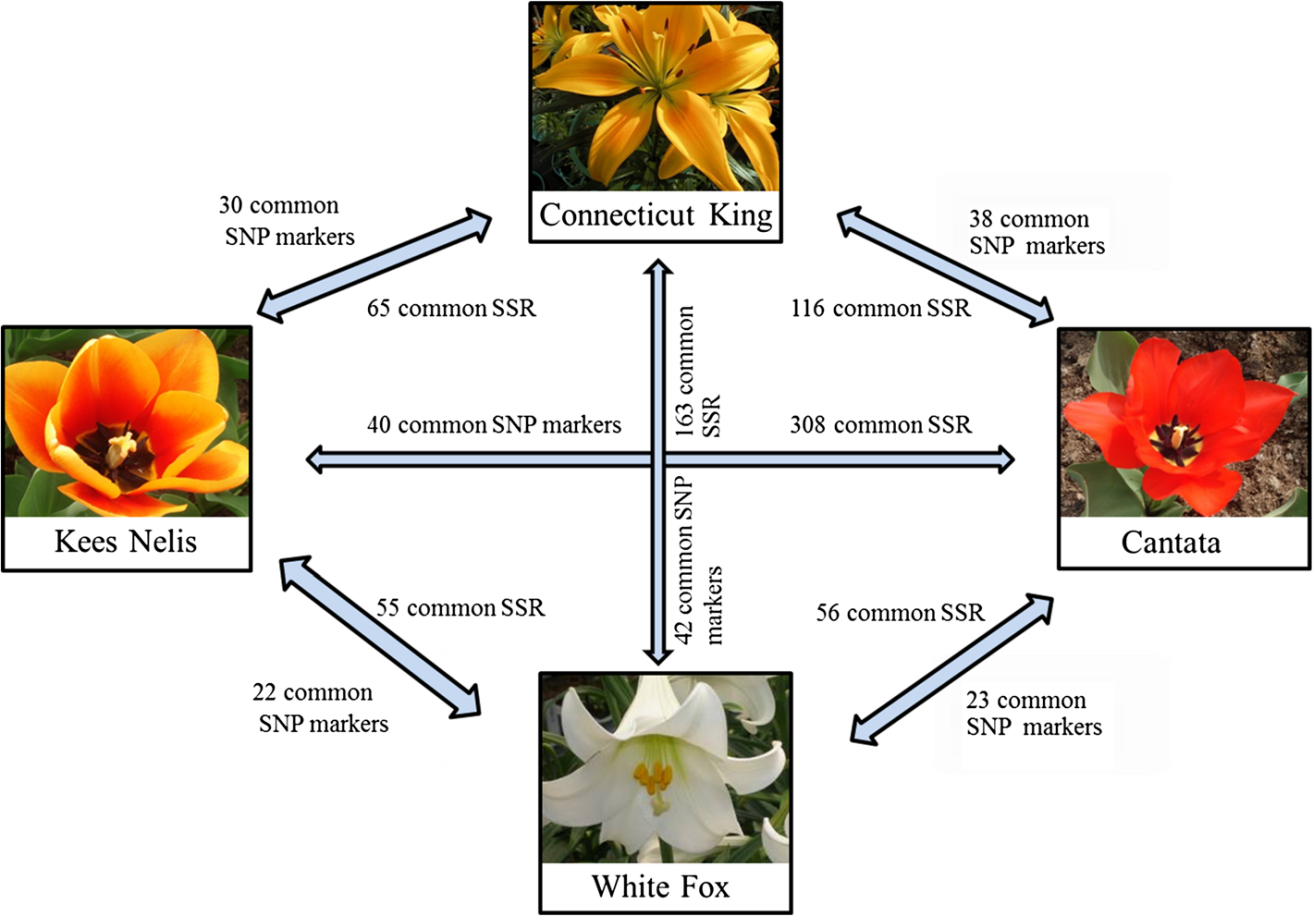
**Common SNP and SSR markers between lily and tulip.** Common markers identified among the parents of the lily and tulip mapping populations developed based on orthologous sequences of each genotype combination. Common markers represent orthologous genetic loci with a polymorphism. Actual polymorphisms may differ between the parents.

As a result, ‘Connecticut King’ showed to have 30 and 38 SNP markers in common with ‘Kees Nelis’ and ‘Cantata’, respectively; ‘White Fox’ has 22 and 23 common SNP markers with ‘Kees Nelis’ and ‘Cantata’, respectively (Figure 4). As for common SSRs, ‘Connecticut King’ showed to have 65 and 116 common EST-SSR with ‘Kees Nelis’ and ‘Cantata’, respectively. Similarly, ‘White Fox’ has 55 and 56 common EST-SSR with ‘Kees Nelis’ and ‘Cantata’, respectively (Figure 4). Thus, 113 common SNP markers and 292 common EST-SSR were identified between the lily and tulip populations. Similarly, common SNP markers between the parents of the lily population and between the parents of the tulip population were identified. ‘Connecticut King’ and ‘White Fox’ have 42 common SNP markers and 163 common EST-SSR; and ‘Cantata’ and ‘Kees Nelis’ have 40 common SNP markers and 308 common EST-SSR (Figure 4).

Efficiency of these markers in a comparative study depends largely upon how many of these markers will be mapped on the genetic maps and also on how well these markers will be distributed over the chromosomes. This also will define if the current number of markers is sufficient to carry out such a synteny study or that more markers should be generated.

## Conclusion

454 pyro-sequencing provides a rich resource for marker development and comparative genomic studies for species with an uncharacterized genome. Large numbers of SNPs amendable for high throughput genotyping purposes were generated for each genotype providing a very rich resource for fast development of markers in lily and tulip. Microsatellites that were mined and characterized for lily and tulip confirmed that there is a selection against di-nucleotide repeats in coding regions while tri-nucleotide repeats were equally spread over coding and UTR regions. Running comparative genomic analysis among lily, tulip, and rice not only identified genes that are shared among these three monocot species, but also identified a set of genes that are present in the two monocot flower bulb species but not in rice. Studying this group of putative specific genes of flower bulbs may provide insight in the biology of these specialized monocots. To improve our understanding of evolutionary history (gene duplication, conversion, rearrangement) of the lily and tulip genomes, we identified common genetic loci with SNP or SSR polymorphisms that can be used as marker in the available mapping populations for lily and tulip.

## Methods

### Plant material

Four lily genotypes that represent the four main hybrid groups of genus *Lilium* were used for sequencing: cv. ‘Star Gazer’ (Oriental, *Archelirion* section), breeding line ‘Trumpet 061099’ (Trumpet, *Leucolirion* section), cv. ‘White Fox’ (Longiflorum, *Leucolirion* section) and cv. ‘Connecticut King’ (Asiatic, *Sinomartagon* section). Five tulip cultivars were used for sequencing: cv. ‘Cantata’ and cv. ‘Princeps’ belonging to *T. fosteriana* (*Eichleres* section) and cv. ‘Bellona’, cv. ‘Kees Nelis’, and cv. ‘Ile de France’ belonging to *T. gesneriana* (*Tulipa* section). Young leaves (500 mg) were collected and kept at −80°C until RNA isolation.

### Methodology

RNA isolation, cDNA library preparation, 454 sequencing procedures, sequence filtrations steps and the assembly using CLC were done as described in Shahin et al. [24]. In brief, cDNA libraries were sequenced on a Life Sciences GS-FLX Titanium according to standard procedures (454 Life Sciences) at Greenomics (Wageningen, the Netherlands).

CLC that uses the De Brijun algorithm was used to assemble the cDNA sequences of lily and tulip [24]. CLC showed to have the capacity to handle sequence data of outcrossing species with heterozygous sequence data [24,26]. Using CLC genomics workbench software (CLC bio, Denmark, http://www.clcbio.com/), the 3′ and 5′ adapter sequences were trimmed. Low quality bases (1 base at the 3′ end and 15 bases from the 5′ end, other low quality terminal bases with a 0.05 threshold) were also removed, and the maximum number of ambiguous nucleotides allowed in the fragment was set to 2. Only fragments between 100–800 bp were kept for further analysis. CD-HIT [54] was used to remove PCR duplicates (clonality) with a threshold of 98% similarity. The *de novo* assembly using CLC was done using the following parameters: conflict resolution (vote), similarity 95%, and alignment mode (global, do not allow InDels).

The contigs (non-redundant sequence) were constructed: for each genotype separately, for the four lily genotypes together (Lily-All), for the five tulip genotypes together (Tulip-All), for *T. fosteriana* cultivars (‘Cantata’ and ‘Princeps’) together, and for *T. gesneriana* cultivars (‘Bellona’, ‘Kees Nelis’, and ‘Ile de France’) together. Contigs of all assemblies (except Lily-All and Tulip-All) were used for transcriptome coverage estimation, SNP marker development, and SSR mining. Lily-All and Tulip-All assemblies were only used for gene annotation and gene ontology.

### Data availability

Raw sequence data of the four cultivars of lily are available at ENA-SRA (European Nucleotide Archive-Sequence Read Archive) with the accession number ERP001106. Raw sequence data of the five cultivars of tulip are available at http://datarelease.plantbreeding.nl/Tulip_Shahin/.

### SNP marker detection

Contigs of single genotype assemblies, and of *T. gesneriana*, and *T. fosteriana* were submitted to an updated version of QualitySNP [28] to detect single nucleotide variants (SNPs). QualitySNP was also used to calculate the number of contigs that contain at least one SNP.

SNP markers were selected twice. The first set was selected based on the following criteria: high quality sequence, not within or adjacent to a homopolymeric tract, at least 2 reads of each allele [26], and 50 bp of flanking sequence on each side. The second set was selected based on the same criteria but here also the presence of a secondary SNP was allowed in the flanking regions. This was done since different high throughput genotyping technologies have different requirements concerning the presence of flanking SNPs. To ensure high quality of the SNP markers, the D value (QualitySNP [28]) was limited to (0–0.5) which reduces the probability that an assembled cluster contains paralogs.

The resulting SNP marker sequences (50 bp flanking the target SNP on each side) of both sets were compared against all contigs using BlastN (1E-20). Only SNP marker sequences which mapped uniquely to the contig from which they were selected were retained [24].

### Mining for microsatellites

Microsatellites were searched using MISA [32] which identifies perfect, compound and interrupted microsatellite motifs. The criteria for selection of microsatellites were a minimum of six repeats for di-nucleotide motifs and five repeats for tri-, tetra-, penta-, and hexa-nucleotide motifs.

Microsatellites positions (coding region or UTRs) for di and tri-nucleotide repeats were identified for each genotype separately. Contigs containing di-nucleotide repeats were collected in one fasta file. Similarly, contigs contain tri-nucleotide repeats were collected in another fasta file. These two fasta files were submitted to ORF-Predictor ( http://proteomics.ysu.edu/tools/OrfPredictor.html, [55]) which tested each contig for the six possible open reading farms and kept only the frame that generated the longest protein. ORF-Predictor subsequently defined open reading frame position, start of coding region, and end of coding region. Next, SSRs are then analyzed for their exact location in the gene with respect to the open reading frame.

### Orthologous sequence

Orthologous and in-paralogous sequences among the four lily genotypes, five tulip genotypes, and rice genome were identified using OrthoMCL [39]. Protein sequences of the rice genome were retrieved from Phytozome ( http://phytozome.net[36]). Only the longest transcript was kept in case more than one variant per locus was present ending with 55,803 rice protein sequences. Information about transposable element related genes in rice were obtained from the rice annotation v 7.0 ( ftp://ftp.plantbiology.msu.edu/pub/data/Eukaryotic_Projects/o_sativa/annotation_dbs/pseudomolecules/version_7.0/all.dir/all.locus_brief_info.7.0). Contigs of the four lily genotypes and five tulip genotypes were translated using ESTscan2 [37,38] using a model pre-trained on rice sequences due to the lack of a species specific codon frequency model.

The 10 sets of protein sequences were the input for the OrthoMCL [39] for the orthologous group prediction. If not otherwise noted, default settings were used. The resulting groups were presented in a Venn diagram [56], in which groups possessing members from all species (lily, rice, and tulip), groups specific to each species, groups specific to lily and tulip, lily and rice, tulip and rice were presented (Figure 1).

### Gene annotation and gene ontology identification

Lily and tulip’s contigs (Lily-All, and Tulip-All) and the orthologous sequences groups (6,900 groups) identified for the three species (lily, rice, and tulip, Figure 1) by OrthoMCL were annotated by blasting (BlastX) to the databases (non-redundant protein sequences-nr) using Blast2Go V.2.4.9 software [45] with an E-value of 1E-15. Blast2Go is an automated tool for the assignment of gene ontology terms and was designed for use with novel sequence data. The distribution of genes in each ontology category was examined and the percentage of unique sequences in each of the assigned GO terms: biological process, molecular function, and cellular component were computed and presented.

### Identification of common SNP and SSR markers within and between the two species

For both species a mapping population is available, in lily, an inter-sectional F1 population (100 progenies) ‘White Fox’ × ‘Connecticut King’ [57] and in tulip, an inter-sectional F1 population (125 progenies) ‘Kees Nelis’ × ‘Cantata’. To link the two species and be able to transfer information from one species to another, common markers that can be mapped in both populations are needed. Common marker in this study refers to markers based on polymorphisms found in the orthologous sequences between and lily and tulip (although the specific polymorphism may differ between the two species), and thus their mapping position can be used to study synteny between the two species. Similarly, common markers within each species (between ‘White Fox’ and ‘Connecticut King’; and between ‘Kees Nelis’ and ‘Cantata’) were identified.

Common marker identification was done based on the orthologous groups generated by OrthoMCL analysis. Only contigs that have one contig of each genotype in the orthologous group (one-to-one relation) were selected to avoid selecting in-paralogs that likely lead to SNP marker dropout in genotyping. Orthologous contigs for each of these genotypes combination: ‘Connecticut King’-‘Whit Fox’ (3,551 contigs each), ‘Connecticut King’-‘Cantata’ (5,590 contigs each), ‘Connecticut King’-‘Kees Nelis’ (2,913 contigs each), ‘Whit fox’-‘Cantata’ (3,024 contigs each), ‘White Fox’-‘Kees Nelis’ (3,611 contigs each), and ‘Kees Nelis’-‘Cantata’ (3,675 contigs) in these one-to-one orthologous relationships were defined. This leads to 6 genotype combinations and 12 orthologous groups (1 for each parent from a genotype combination).

These 12 orthologous groups were submitted to QualitySNP for SNP marker identification using the following criteria: 50 bp flanking regions, one secondary SNP in flanking regions was allowed, D value 0–0.5, and only one SNP per contig was selected.

To select only orthologous contigs that generate SNP markers in both genotypes of genotype combinations, identified SNP marker contigs for each genotype were blasted against each other (E-5).

To identify SSR markers in these combinations, the same sets of orthologous contigs were assembled together, and then used for SSR identification using MISA [32] applying the same criteria explained previously. Assembly of theses orthologous improves SSR detection since it might increase contigs length, and thus increase the chance of designing primers.

## Competing interests

The authors declare that they have no competing interests.

## Authors’ contributions

MvK performed the CLC assembly, DE performed SSR mining. JWB performed OrthoMCL and Venn diagram analysis. AS performed SNP identification by QualitySNP, and all the comparisons mentioned in this study in addition to the gene annotation and gene ontology parts. PA and AS participated in the interpretation of the results and writing the manuscript. JvT and RGFV participated in the coordination of the study. All authors read and proved the final manuscript.

## Supplementary Material

Additional file 1 **Gene annotation and gene ontology for the lily contigs generated by blasting contigs to the nr genebank with a threshold of 1E-15 using Blast2go software.** GO terms: F stands for molecular function, P for biological process, and C for cellular compound. Click here for file

Additional file 2 **Gene annotation and gene ontology for the tulip contigs generated by blasting contigs to the nr genebank with a threshold of 1E-15 using Blast2go software.** GO terms: F stands for molecular function, P for biological process, and C for cellular compound. Click here for file

Additional file 3 **Gene annotation and gene ontology for the orthologous groups between lily, tulip, and rice generated by blasting contigs to the nr genebank with a threshold of 1E-15 using Blast2go software.** GO terms: F stands for molecular function, P for biological process, and C for cellular compound. Click here for file
